# Antifungal Siderophore Conjugates for Theranostic Applications in Invasive Pulmonary Aspergillosis Using Low-Molecular TAFC Scaffolds

**DOI:** 10.3390/jof7070558

**Published:** 2021-07-14

**Authors:** Joachim Pfister, Milos Petrik, Katerina Bendova, Barbara Matuszczak, Ulrike Binder, Matthias Misslinger, Alexander Kühbacher, Fabio Gsaller, Hubertus Haas, Clemens Decristoforo

**Affiliations:** 1 Department of Nuclear Medicine, Medical University Innsbruck, A-6020 Innsbruck, Austria; joachim.pfister@i-med.ac.at; 2 Institute of Molecular and Translational Medicine, Faculty of Medicine and Dentistry, Palacky University Olomouc, 77200 Olomouc, Czech Republic; milos.petrik@upol.cz (M.P.); katerina.bendova01@upol.cz (K.B.); 3 Institute of Pharmacy/Pharmaceutical Chemistry, University of Innsbruck, A-6020 Innsbruck, Austria; Barbara.matuszczak@uibk.ac.at; 4 Institute of Hygiene & Medical Microbiology, Medical University of Innsbruck, A-6020 Innsbruck, Austria; ulrike.binder@i-med.ac.at; 5 Institute of Molecular Biology, Medical University of Innsbruck, A-6020 Innsbruck, Austria; matthias.misslinger@i-med.ac.at (M.M.); alexander.kuehbacher@i-med.ac.at (A.K.); fabio.gsaller@i-med.ac.at (F.G.); hubertus.haas@i-med.ac.at (H.H.)

**Keywords:** siderophore, TAFC, antifungal, invasive pulmonary aspergillosis, *Aspergillus fumigatus*, antifungal susceptibility testing, PET/CT, theranostics

## Abstract

Invasive pulmonary aspergillosis (IPA) is a life-threatening form of fungal infection, primarily in immunocompromised patients and associated with significant mortality. Diagnostic procedures are often invasive and/or time consuming and existing antifungals can be constrained by dose-limiting toxicity and drug interaction. In this study, we modified triacetylfusarinine C (TAFC), the main siderophore produced by the opportunistic pathogen *Aspergillus fumigatus* (*A. fumigatus*), with antifungal molecules to perform antifungal susceptibility tests and molecular imaging. A variation of small organic molecules (eflornithine, fludioxonil, thiomersal, fluoroorotic acid (FOA), cyanine 5 (Cy5) with antifungal activity were coupled to diacetylfusarinine C (DAFC), resulting in a “Trojan horse” to deliver antifungal compounds specifically into *A. fumigatus* hyphae by the major facilitator transporter MirB. Radioactive labeling with gallium-68 allowed us to perform in vitro characterization (distribution coefficient, stability, uptake assay) as well as biodistribution experiments and PET/CT imaging in an IPA rat infection model. Compounds chelated with stable gallium were used for antifungal susceptibility tests. [Ga]DAFC-fludioxonil, -FOA, and -Cy5 revealed a MirB-dependent active uptake with fungal growth inhibition at 16 µg/mL after 24 h. Visualization of an *A. fumigatus* infection in lungs of a rat was possible with gallium-68-labeled compounds using PET/CT. Heterogeneous biodistribution patterns revealed the immense influence of the antifungal moiety conjugated to DAFC. Overall, novel antifungal siderophore conjugates with promising fungal growth inhibition and the possibility to perform PET imaging combine both therapeutic and diagnostic potential in a theranostic compound for IPA caused by *A. fumigatus*.

## 1. Introduction

Invasive aspergillosis (IA) is a severe infection in humans, associated with high mortality and an estimated incidence of 250,000 cases a year worldwide [1,2]. IA most commonly involves the lungs resulting in invasive pulmonary aspergillosis (IPA) in immunocompromised patients with *Aspergillus fumigatus* (*A. fumigatus*) as the most common opportunistic pathogen involved [3,4].

Diagnostic procedures recommended by current guidelines [5] can be invasive (e.g., bronchoalveolar lavage, bronchoscopy) and/or time consuming, and can worsen the survival outcome for patients [2,6]. Current treatment options are azoles, echinocandins, and polyenes [5,7], but antifungal resistance is a concern for the management of *A. fumigatus* infections [8]. Furthermore, antifungal agents are often constrained by drug interaction, route of administration, and dose-limiting toxicity. These factors are very alarming and demand for a continuous improvement in the diagnosis and treatment of IPA.

During infection, the human body sequesters iron as a defense strategy to prevent the growth of pathogens [9]. Iron is an essential micronutrient and *A. fumigatus* has developed sophisticated strategies to overcome this problem: low-affinity iron uptake, reductive iron assimilation (RIA), and siderophore-mediated iron acquisition (SIA) [10], whereby RIA and SIA are high-affinity iron uptake systems. SIA is highly upregulated during infection and essential for the virulence of *A. fumigatus* [11]. Siderophores are low-molecular mass organic molecules with a high affinity to bind ferric iron (Fe(III)). *A. fumigatus* produces different types of siderophores: desferri-fusarinine C (FsC) and desferri-triacetylfusarinine C (TAFC) for external iron acquisition, and desferri-ferricrocin and desferri-hydroxy-ferricrocin for internal iron storage [12]. After binding iron in the environment, [Fe]TAFC is taken up by the hyphae with a specific transporter called MirB [13]. This transporter is highly upregulated during infection [11] and, additionally, [Fe]TAFC shows a very high specificity for *A. fumigatus* lacking uptake by mammalian cells [14,15]. This offers an opportunity to use MirB as a target for imaging and treatment of *A. fumigatus* infections.

**Figure 1:**
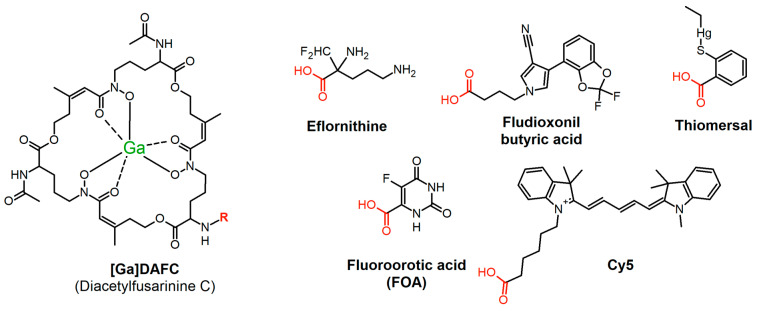
Chemical structures of antifungal compounds used in this study. On the left side: gallium-labeled form of diacetylfusarinine C (DAFC); red part of the structures indicate coupling site of individual molecules.

Petrik et al. showed the possibility of exchanging iron from [Fe]TAFC with the radioactive isotope gallium-68 to perform PET/CT imaging in an lung infection animal model [16]. Furthermore, by using diacetylfusarinine C ([Fe]DAFC), which possesses a free amine group, chemical modifications are possible [17]. In previous experiments by our group, we used fluorescent dyes coupled to DAFC to perform fluorescence microscopy of *A. fumigatus* hyphae [18] labeled with gallium-68, and we performed hybrid imaging of an infection in a rat model [19]. This proof of principle using TAFC as a “Trojan horse” to channel a variety of molecules specifically into *A. fumigatus* via the MirB transporter, led us to the idea to adopt this system for antifungal molecules. For this purpose, different candidates (Figure 1) were chosen depending on certain, individual properties.

Eflornithine, an inhibitor of ornithine decarboxylase [20], is a multifunctional drug as it is used topically (15% eflornithine cream) against unwanted facial hair (hirsutism) [21], as a treatment for malignant gliomas [22], and as an intravenous cocktail in combination with nifurtimox, approved by the EMA against African trypanosomiasis (sleeping sickness) caused by *Trypanosoma brucei gambiense* [23,24]. Beckmann et al. showed an antifungal activity against *A. fumigatus* in agar diffusion assays [25], which potentially could be enhanced by active transport into the hyphae via MirB. The same rational was applied for fluoroorotic acid, which is converted by ornithine-5′-monophosphate (OMP)-decarboxylase to the toxic intermediate 5-fluoro-UMP, commonly used for selection in yeast genetic experiments [26]. Furthermore, we chose fludioxonil, a phenylpyrrole pesticide used post-harvest for fruit and vegetable crops to minimize losses from mold contamination during transport and point of sale. It inhibits class III hybrid histidine kinases (HHK) that are peculiar to fungi and regulate the high osmolarity glycerol pathway (HOG) [27]. For the last 30 years it has been postulated to be safe for human as there is no target in the body, but studies are currently ongoing to investigate if there are adverse health effects on the cellular level [28]. By coupling fludioxonil to DAFC, the active uptake by MirB could lower the administered dosage needed and interaction with human targets could be hindered by this modification. A similar concept could be applied for thiomersal, which was commonly used as a preservative substance in vaccines and ophthalmic products [29,30], but adverse health effects of ethylmercury (active metabolite) are still controversially discussed [31]. The last compound Cy5 was originally designed as an agent for hybrid imaging and microscopy, whereby an antifungal activity was observed during growth experiments by our group [18,19].

The aim of the study was to investigate the growth inhibition potential of various antifungal siderophore conjugates and to visualize an *A. fumigatus* lung infection via PET/CT in a rat model by labeling the compounds with gallium-68. Thereby, beside antifungal properties, pharmacokinetic differences of individual compounds were also revealed, providing essential data for further applications aiming at MirB as a drug target. This proof of principle shows the possibility of using these compounds for therapy and diagnostics with the same molecule, in a so called theranostic approach.

## 2. Materials and Methods

### 2.1. Chemicals and Synthesis of Antifungal Siderophore Conjugates

All chemicals were purchased from Sigma Aldrich (Steinheim, Germany) as reagent grade and used without further purification unless stated otherwise. Exact synthetic procedures and analytical details can be found in the Supplementary Materials. Starting from iron-containing diacetylfusarinine C ([Fe]DAFC), all antifungals were conjugated forming an amide bond using *O*-(7-Azabenzotriazol-1-yl)-*N*,*N*,*N*′,*N*′-tetramethyluronium hexafluorophosphate (HATU) as an activating reagent. To couple eflornithine, the free amino groups in the molecular structure first were protected with tert-butyloxycarbonyl (Boc) before linking with [Fe]DAFC, followed by the cleavage of the Boc-protecting groups with trifluoroacetic acid. Fludioxonil had to be modified with ethyl 4-bromobutyrate resulting in a new compound with an ester function, followed by hydrolysis to get the free carboxylic acid, suitable for HATU coupling (analytical data see Figures S1 and S2).

All antifungal siderophore conjugates were purified by preparative HPLC (Gilson, Middleton, WI) and freeze dried. For iron removal, compounds were incubated with 100 mM Na_2_EDTA solution and subsequently purified by preparative HPLC, resulting in the iron-free form for radiolabeling experiments. Stable gallium-containing antifungal siderophore conjugates was produced by coupling the antifungal directly to [Ga]DAFC to reduce iron contamination to a minimum. Exact coupling conditions and a detailed description can be found in the supplementary material as well as analytical data for each compound, respectively.

### 2.2. Radiolabeling

Gallium-68 was produced by fractionated elution of ^68^Ge/^68^Ga-generator (IGG100. Eckert and Ziegler Isotope Products, Berlin, Germany; nominal activity of 1850 MBq) with 0.1 M hydrochloric acid (HCL, Rotem Industries, Arva, Israel). For labeling, 10 μg (5–8 nmol) of DAFC conjugate was mixed with 200 μL gallium eluate (~15–30 MBq) and the pH was adjusted to 4.5 by adding 20 μL of sodium acetate solution (1.14 M) per 100 μL eluate. The mixture was left to react for 10 min at RT and finally analyzed by radio-TLC (Dionex, Germering, Germany) and radio-RP-HPLC (Gabi Star, Raytest; Straubenhardt, Germany) [32].

### 2.3. In Vitro Experiments

#### 2.3.1. Distribution Coefficient (LogD)

To determine the lipophilicity of the antifungal siderophore conjugates, the distribution coefficient between octanol and PBS buffer was determined. Radiolabeled compounds were dissolved in PBS to a concentration of approximately 9 µM and 50 µL of this solution was added to 450 μL PBS and 500 μL octanol into an eppendorf tube. The two phases were vigorously shaken for 20 min at 1400 rpm at room temperature (MS 3 basic vortexer, IKA, Staufen, Germany) followed by centrifugation for 2 min at 4500 rpm (Eppendorf Centrifuge 5424, Eppendorf AG, Hamburg, Germany).

Hereafter, 200 µL of each phase was collected and measured in a 2480 automatic Gamma counter Wizard 2 3″ (PerkinElmer, Waltham, MA, USA). LogD values were calculated using Excel by dividing measured values of octanol by PBS and logarithmizing the result. Values > 0 reflect lipophilic, values < 0 reflect hydrophilic compounds (*n* = 3, six technical replicates).

#### 2.3.2. Protein Binding

For this procedure antifungal siderophore conjugates were labeled as described before and diluted with PBS to a concentration of approximately 9 µM. Next, 50 µL of this solution was added to 450 µL of PBS (control) or 450 µL of fresh human serum and incubated at 37 °C for 30, 60, and 120 min. At each timepoint, 25 µL of PBS/serum was analyzed by size exclusion chromatography using MicroSpin G-50 columns (Sephadex G-50, GE Healthcare, Vienna, Austria) according to the manufacturer’s protocol. Hereafter, the column and eluate were measured separately in the gamma counter and calculated by dividing measured counts of eluate by total counts and multiplied by 100, resulting in percentage of protein bound conjugate. Radioactivity in the eluate reflects the protein bound fraction and column bound, free-labeled siderophore conjugate (*n* = 3, three technical replicates).

#### 2.3.3. Serum Stability

Serum stability probes were prepared according to protein-binding section with PBS and fresh human serum. After 60, 120, and 240 min, 70 µL of serum/PBS was mixed with 70 µL of acetonitrile to precipitate proteins in the serum. Hereafter, the mixture was centrifuged for 1 min and 70 µL of the supernatant was diluted with water and analyzed by radio-HPLC to determine the intact-labeled siderophore conjugate (*n* = 2, two technical replicates).

#### 2.3.4. Uptake and Competition Assay

Uptake assays were performed as previously described [17]. Cultures were prepared by incubation of *A. fumigatus* conidia (1 × 10^6^ conidia/mL) in 200 mL liquid iron-depleted and iron-replete aspergillus minimal media (AMM) [33] for 20 h at 37 °C and constant shaking. Next, 180 µL of the liquid culture was added in pre-wetted 96-well MultiScreen Filter Plates HTS (1 μm glass fiber filter, Merck Millipore, Darmstadt, Germany) and incubated for 15 min at 37 °C with either 25 µL PBS or 25 µL [Fe]TAFC solution (control—uptake block; ~10 µM). Hereafter, 50 µL of radiolabeled compound (final concentration approximately 90 nM) was added and incubated for another 45 min at 37 °C. Hyphae were washed two times with ice-cold TRIS buffer and dry filters were measured in the gamma counter.

Competition assays were performed in the same way except that fungal cultures were pre-incubated with iron-labeled antifungal siderophore conjugates (~10 µM) for 15 min and the uptake value of [^68^Ga]Ga-TAFC into hyphae was determined in order to demonstrate specific interaction with the MirB transporter by the reduction of uptake of [^68^Ga]Ga-TAFC.

#### 2.3.5. Growth Promotion Assay

This procedure was performed as previously described [34]. In this assay, a mutant strain of *A. fumigatus* (*ΔsidA/ΔftrA*) that lacks the genes *sidA* and *ftrA* was used. These mutations impair both siderophore biosynthesis and reductive iron acquisition (RIA), in other words, endogenous high-affinity iron acquisition [11]; however, this mutant is still able to take up siderophores. Consequently, utilization of siderophores can be simply tested by growth promotion assays allowing us to analyze the effect of siderophore modification compared to the original molecule such as [Fe]TAFC. In case of growth reduction, distinction between reduced iron utilization and antifungal activity is not possible. Conidia (10^4^) were point inoculated on solid 0.5 mL of iron-depleted AMM in 24-well plates containing increasing concentrations of iron-containing siderophore ranging from 0.1 to 50 μM. Plates were incubated for 48 h at 37 °C in a humidity chamber and visually assessed [19] (*n* = 3, three biological replicates).

#### 2.3.6. Antifungal Susceptibility Assays

Antifungal susceptibility assays were performed with 96-well flat bottom plates (Greiner Bio-One GmbH) prepared with 100 μL of iron-depleted [Fe(−)] and iron-replete [Fe(+)] 2 × AMM containing 3 × 10^4^ spores of *A. fumigatus* (ATCC 46645) per well.

Next, 100 µL of antifungal siderophore conjugate, dissolved in water, was added to get a final concentration of 1 × AMM and antifungal siderophore conjugates in serial 2-fold dilutions in concentrations ranging from 256 to 0.016 µg/mL. The mtinimal inhibitory concentration (MIC) value was defined as the lowest concentration resulting in no visible fungal growth after 24 h and 48 h incubation at 37 °C in a humidity chamber. Assays were repeated three times as biological replicates (*n* = 3).

Results were also displayed by taking microscopy pictures of each well after 24 h. Images were acquired with the IncuCyte S3 Live-Cell Analysis System equipped with a 20× magnification S3/SX1 G/R Optical Module (Essen BioScience Inc., Hertfordshire, UK). From each well a representative image was taken from the center of the well. Fungal growth was analyzed using the Basic Analyzer tool (Confluence%; Segmentation adjustment: 0; Adjust Size: 0) of the IncuCyte S3 software (Version 2019; Essen Bioscience Inc.). Images and the confluence mask were exported in raw 8-bit images and a raw 8-bit confluence mask, respectively. An overview of these pictures can be seen in the Supplementary Materials (Figures S3 and S4).

### 2.4. Animal Experiments

All animal experiments were conducted in compliance with the Austrian and Czech Animal Protection laws and with approval of the Austrian Ministry of Science (BMWFW-66.011/0161-WF/V/3b/2016), the Czech Ministry of Education Youth and Sports (MSMT-21275/2016-2), and the institutional Animal Welfare Committee of the Faculty of Medicine and Dentistry of Palacky University in Olomouc.

#### 2.4.1. In Vivo Stability and Ex Vivo Biodistribution

Stability test and biodistribution were conducted in 4–6-week-old female BALB/c mice (in-house breed, ZVTA Innsbruck). ^68^Ga-labeled antifungal siderophore conjugates were injected via lateral tail vein using approximately 0.4 nmol of siderophore conjugate.

In vivo stability was determined by radio-HPLC analysis. After injection of the radiolabeled compound (~12 MBq) the mouse was euthanized after 10 min by cervical dislocation. Blood was collected and precipitated with ACN to remove proteins. Subsequently, the supernatant was diluted with water and used for further analysis. Urine samples were directly injected into the radio-HPLC. Percentage of intact radiolabeled siderophore conjugate was calculated by integration of the radio-chromatogram (*n* = 2) [35].

For biodistribution, a similar procedure was applied, but the mice were euthanized at 45 and 90 min. Hereafter, organs (blood, spleen, pancreas, stomach, liver, kidneys, heart, lung, muscle, femur) were removed and weighted. Samples were measured in a gamma counter and results expressed as percentage of injected dose per gram tissue (%ID/g). (*n* = 3)

#### 2.4.2. Invasive Pulmonary Aspergillosis Model in Rats

Additionally, 2–3-month-old female Lewis rats were treated with the immunosuppressant cyclophosphamide (Endoxan, Baxter, Prague, Czech Republic, 75 mg/kg i.p.) 5 days and 1 day before they were infected with *A. fumigatus*, to induce neutropenia. The animals repeatedly received (5 days, 1 day before, and on the day of inoculation) the antibiotic teicoplanin (Targocid, Sanofi-Aventis, Prague, Czech Republic, 35 mg/kg—5 days before i.m., or 25 mg/kg i.m.—1 day before and on the day of inoculation) to avoid bacterial superinfections, and additional antibiotics were administered by drinking water (Ciprofloxacin, Fresenius Kabi, Prague, Czech Republic, 2 mM, Colomycin, Teva, Prague, Czech Republic, 0.1 mM) for the duration of the experiment. Infection in the lung was established by intratracheal inoculation of 100 μL of *A. fumigatus* spores (10^9^ CFU/mL *A. fumigatus* ATCC 46645) using TELE PACK VET X LED system equipped with a flexible endoscope (Karl Stroz GmbH and Co. KG, Tuttlingen, Germany) [36].

#### 2.4.3. PET/CT Imaging

In vivo PET/CT imaging was conducted 2–4 days after *A. fumigatus* inoculation, depending on the developed infection and health condition of the animal. Rat PET/CT images were acquired with an Albira PET/SPECT/CT small animal imaging system (Bruker Biospin Corporation, Woodbridge, CT, USA). Radiolabeled antifungal siderophore conjugates were administered by retro-orbital (r.o.) injection of a 5–10 MBq dose corresponding to ~2 μg of the DAFC conjugate per rat.

Animals were anaesthetized with isoflurane (Forane^®^, Abbott Laboratories, Abbott Park, IL, USA) (2% flow rate) and positioned prone headfirst in the Albira system before the start of imaging. Static PET/CT imaging was carried out 45 min p.i. for all tested compounds. A 10-min PET scan (axial FOV 148 mm) was performed, followed by a triple CT scan (axial FOV 3 × 65 mm, 45 kVp, 400 μA, at 400 projections). Scans were reconstructed with the Albira software, v. 0,900,111 5.8 (Bruker Biospin Corporation, Woodbridge, CT, USA) using the maximum likelihood expectation maximization (MLEM) and filtered backprojection (FBP) algorithms. After reconstruction, acquired data were viewed and analyzed with PMOD software, v. 3.307 (PMOD Technologies Ltd., Zurich, Switzerland).

## 3. Results

### 3.1. Synthesis and Radiolabeling

The precursor preparation of eflornithine, made by shielding the free amino groups with Boc protection, was obtained in a moderate yield (>20%, non-optimized) but was suitable for conjugation to [Fe]/[Ga]DAFC with standard HATU coupling. Synthesis of fludioxonil-butyric acid was carried out using a two-step reaction, which involved *N*-alkylation of the pyrrole with ethyl 4-bromobutyrate and was followed by basic hydrolysis of the ethyl ester group. Hereafter, the free carboxylic acid could easily couple to the [Fe]/[Ga]DAFC siderophore with very good yields over 60%. All other conjugates were directly linked by our standard HATU coupling strategy with yields of 20–80%. Exact analytical data are provided in the Supplementary Materials.

Radiolabeling of all compounds was achieved with almost quantitative radiochemical yields (>95%) in 10 min at room temperature. Labeled compounds were used without any further purification.

### 3.2. In Vitro Characterization

#### 3.2.1. LogD, Protein Binding, and Serum Stability

The distribution coefficient (LogD), protein binding, and serum stability in fresh human serum of all antifungal siderophore conjugates are summarized in Table 1. LogD values ranging from −3.45 to 1.30 revealed heterogeneous solubility properties of conjugated antifungals compared to LogD of the precursor molecule [Fe]DAFC with −2.34 [17].

Protein binding revealed overall low and consistent values over time, except for [^68^Ga]Ga-DAFC-thiomersal, which was ~70%. Compared to [^68^Ga]Ga-DAFC-FOA with an even higher LogD of 1.3 but lower protein binding (~4%), this could be explained by the reduced serum stability of [^68^Ga]Ga-DAFC-thiomersal, which was ~80%. All other conjugates revealed a high stability in human serum, even after 240 min.

#### 3.2.2. Uptake and Competition Assay

**Figure 2:**
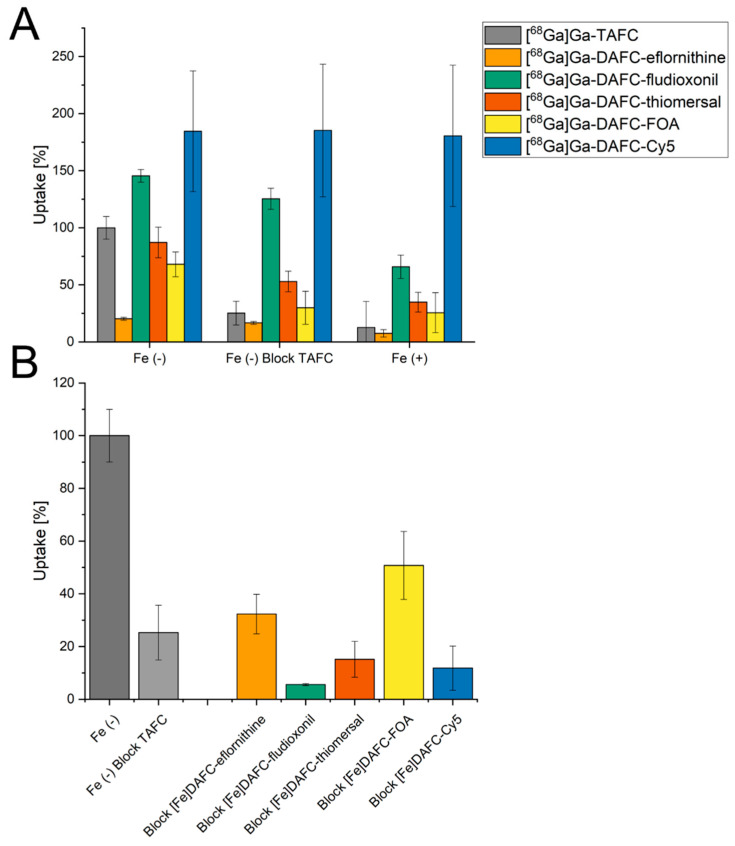
(**A**) Uptake of radiolabeled antifungal siderophore conjugates normalized to reference uptake of [^68^Ga]Ga-TAFC of each experiment, respectively. Grey bars represent control with [^68^Ga]Ga-TAFC. Blocking experiments with [Fe]TAFC due to competition at the MirB transporter should reduce uptake. Iron-replete conditions [Fe (+)] lead to a downregulation of MirB biosynthesis and active uptake of siderophores into the hyphae. (**B**) Competition assay of [^68^Ga]Ga-TAFC blocked with iron-containing antifungal siderophore conjugates in iron-depleted fungal culture. For all compounds a reduction of [^68^Ga]Ga-TAFC uptake could be observed, indicating a specific interaction with the MirB transporter. Data of DAFC-Cy5 adopted from reference [18].

Uptake and competition assays are summarized in Figure 2. Data of DAFC conjugates are normalized to [^68^Ga]Ga-TAFC value of each experiments, respectively, to minimize biological variance. Uptake blocking by [Fe]TAFC results in a competition at the MirB transporter and should decrease the uptake. Under iron-replete conditions, MirB transporter is repressed at transcriptional level [37] and, therefore, the “uptake” under this condition represents an unspecific uptake. All compounds showed reasonable values, i.e., although not as pronounced as found for [^68^Ga]Ga-TAFC, except for [^68^Ga]Ga-DAFC-Cy5 with very high unspecific binding in comparison to [^68^Ga]Ga-TAFC, which could not be reduced during blocking and iron-sufficient conditions. [^68^Ga]Ga-DAFC-fludioxonil also revealed higher values than [^68^Ga]Ga-TAFC, but the uptake reduction was possible by blocking and even more under iron-replete conditions, indicating a MirB-dependent uptake. [^68^Ga]Ga-DAFC-eflornithine, -thiomersal, and -FOA showed a similar pattern; however, eflornithine unveiled only 20% uptake.

In the ferric form, all antifungal siderophore conjugates were able to block uptake of [^68^Ga]Ga-TAFC in competition experiments, confirming interaction with the MirB transporter. All compounds showed comparable or even better blocking values compared to [Fe]TAFC.

#### 3.2.3. Growth Promotion Assay

**Figure 3:**
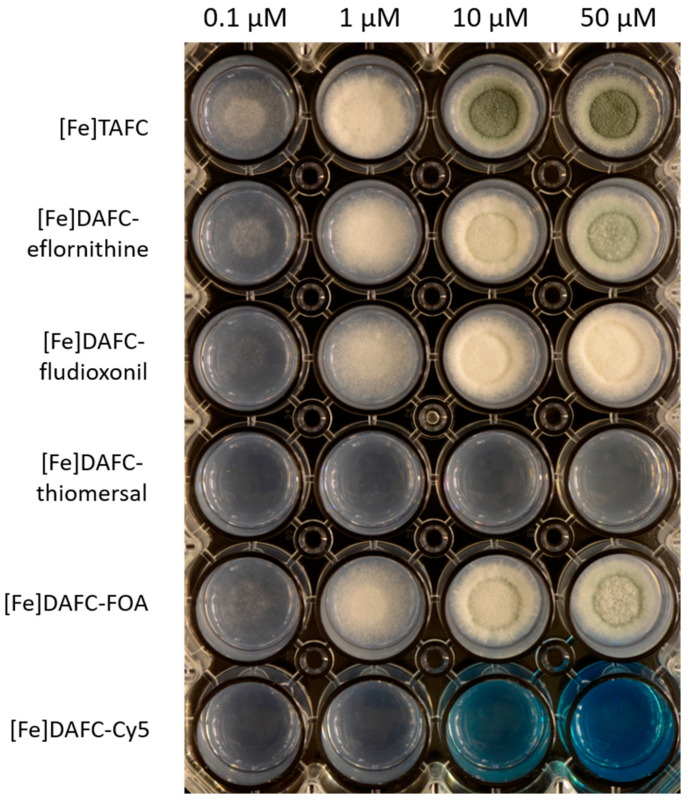
Growth promotion of *A. fumigatus* mutant strain *∆sidA/∆ftrA* after 48 h incubation at 37 °C on iron-depleted AMM with different concentrations of iron-containing antifungal siderophore conjugates. Hyphal growth can be distinguished from greenish (sporulation) and whitish (sterile) mycelia.

Growth promotion of the mutant strain of *A. fumigatus* (*ΔsidA/ΔftrA*) by [Fe]DAFC-conjugates is shown in Figure 3. Control with [Fe]TAFC showed a growth induction at 0.1 µM and sporulation at 10 µM, seen by the green-colored conidia of *A. fumigatus*. Similar growth as in [Fe]TAFC-containing media could be observed for [Fe]DAFC-eflornithine, -fludioxonil, and -FOA, whereby with the eflornithine and FOA conjugates, sporulation was shown at 50 µM. Both [Fe]DAFC-thiomersal and -Cy5 showed no growth promotion at all, indicating that iron from these conjugates cannot be utilized. It should also be considered that there is a competition between iron-induced growth and a growth inhibitory effect of Cy5, but the positive impact of iron is only present if the fungus can utilize iron from the siderophore. This limitation should be kept in mind.

#### 3.2.4. Antifungal Susceptibility Assays

**Figure 4:**
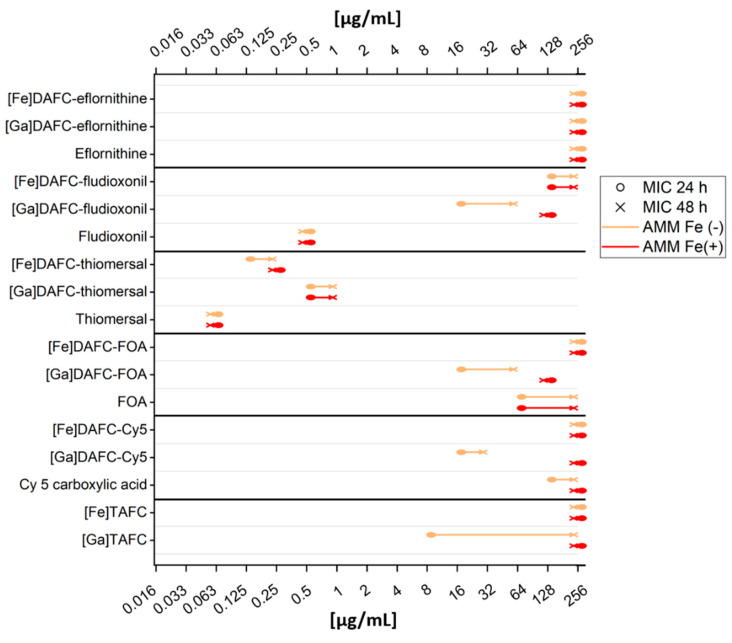
Scheme of all MIC results presented in this study. Orange lines present MIC values with iron-depleted medium (overexpression of the MirB transporter). Red lines are with iron-sufficient medium, which downregulates uptake by siderophore transporters such as MirB. Circle dot of the line displays 24 h and the “×” dot displays 48 h. A table with all data points is included in the Supplementary Materials (Table S1).

Antifungal susceptibility assays were performed to evaluate the antifungal potential of the newly synthesized conjugates. Assays were performed as described in the Methods section, with defining the MIC as the lowest concentration resulting in no visible growth seen by the naked eye. A graphic overview is shown in Figure 4 and a list of all data is included in the Supplementary Materials (Table S1). Most iron-containing compounds showed a higher MIC value compared to their gallium-chelated counterparts, except for DAFC-eflornithine and -thiomersal, which can be explained by the growth promoting effect of iron during iron starvation. Since gallium has an antifungal effect by itself [38], [Ga]TAFC is shown as a control, but growth inhibition did not persist up to 48 h. Thiomersal conjugates showed the strongest growth inhibition, but there was no difference to iron-sufficient media (MirB suppression) or to the original molecule, indicating that antifungal activity is not dependent on active uptake by the fungus. [Ga]DAFC-fludioxonil, -FOA, and -Cy5 showed promising MIC values, that were higher in iron-sufficient media (indicating MirB dependence) and lower than the original molecule (enhanced antifungal effect due to active uptake) except for fludioxonil. Chemical modification of the original molecule fludioxonil (0.5 µg/mL, 24 h) reduced the antifungal effect (fludioxonil butyric acid: 32 µg/mL, 24 h see Table S1), but the MIC value of the modified molecule was still preserved after conjugation. Eflornithine revealed no antifungal effect according to the definition of no visual growth, but still, a growth-reducing effect was observed. Microscopic pictures of all MIC results at 24 h can be seen in the Supplementary Materials (Figures S3 and S4).

### 3.3. In Vivo Experiments

#### 3.3.1. In Vivo Stability and Biodistribution

Results of in vivo stability of the different compounds were heterogeneous and are shown in Table 2. [^68^Ga]Ga-DAFC-eflornithine and -FOA showed a very high stability both in blood and urine samples. [^68^Ga]Ga-DAFC-Cy5 showed no degradation in the blood sample, but there was almost no intact conjugate after excretion through the urinary tract. The last two compounds [^68^Ga]Ga-DAFC-fludioxonil and -thiomersal revealed a high instability in both tested compartments. Only for the thiomersal conjugate was instability also observed in the in vitro serum stability tests.

Biodistribution after 45 min and 90 min of antifungal siderophore conjugates are shown in Table 3. The main excretion of [^68^Ga]Ga-DAFC-eflornithine seemed to be the urinary tract with retention in the kidneys of around 20%, comparable to its high hydrophilicity and stability. On the contrary, [^68^Ga]Ga-DAFC-fludioxonil showed an accumulation in the intestine of the mouse and therefore indicated hepatobiliary excretion. Very high blood levels were found for [^68^Ga]Ga-DAFC-thiomersal, which correlated with the protein-binding values found in vitro. Accumulation in the intestine and kidney retention was also found for this compound. Similar to these results, [^68^Ga]Ga-DAFC-Cy5 showed overall higher blood levels, but also an increasing accumulation of compound in the intestine (15–24%; 45–90 min) as well as in the liver (20–23%). This could be a limitation for PET imaging due to a higher background signal. [^68^Ga]Ga-DAFC-FOA showed no significant accumulation in any organ over time.

#### 3.3.2. PET/CT Images

**Figure 5:**
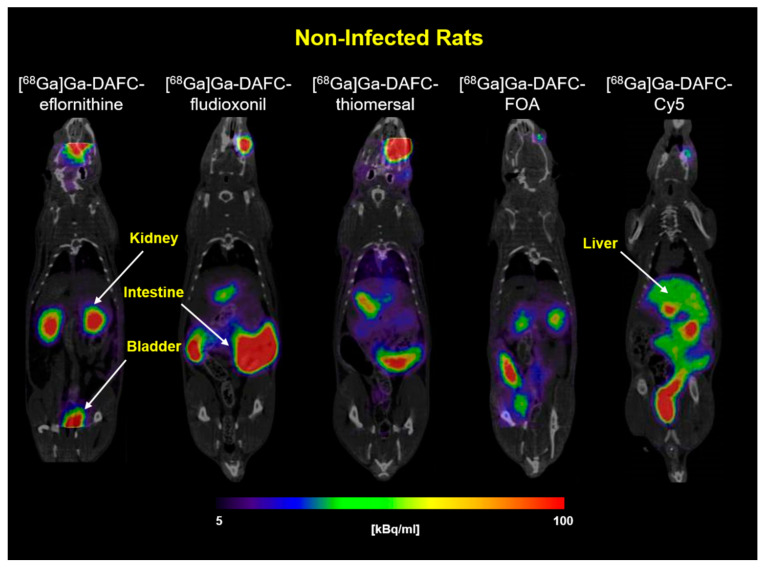
Coronal PET/CT images of non-infected Lewis rats after 45 min post injection of ^68^Ga-labeled antifungal siderophore conjugates (approx. 5–10 MBq injected dose). Kidneys, intestine, liver, and bladder are highlighted by arrows in the picture. Radioactive spots in the eye region originate from the retro-orbital injection.

In Figure 5, coronal PET/CT slices of non-infected Lewis rats are shown, injected with different ^68^Ga-labeled antifungal siderophore conjugates. [^68^Ga]Ga-DAFC-eflornithine and -FOA showed a primary excretion through the urinary tract with clear delineation of the kidneys and bladder. As already described in the biodistribution experiments, [^68^Ga]Ga-DAFC-fludioxonil, -thiomersal, and -Cy5 were excreted through the hepatobiliary way, which resulted in a high signal mainly in the intestinal region.

**Figure 6:**
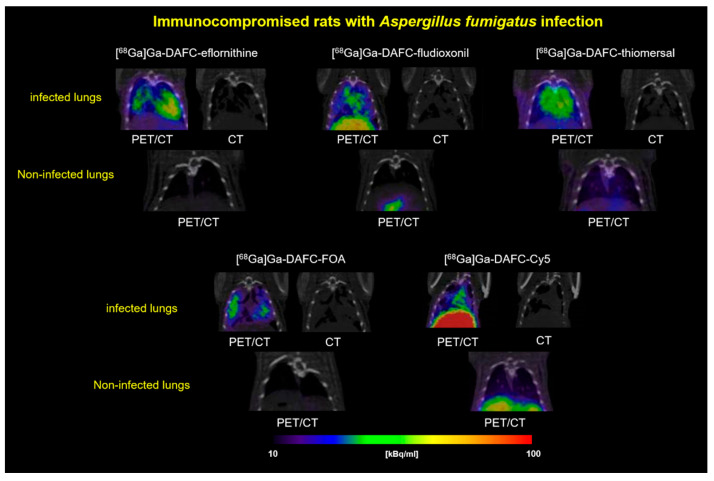
Coronal PET/CT slices of immunocompromised Lewis rats infected with *A. fumigatus* in the lung. Images are showing the lung section of infected (top row) and non-infected (bottom row, control) rats of each compound, respectively. Animals were injected retro-orbitally and images were made after 45 min with approx. 5–10 MBq injected dose. CT images were added to show the severity of the infected lung tissue.

PET/CT images of *A. fumigatus* lung-infected Lewis rats displayed an accumulation (higher or lower) of all antifungal siderophore conjugates in the infected region (Figure 6). CT images of the lung revealed anatomical changes with co-localization of radioactive signal. Skriba et al. showed that these anatomical changes originate from infection with *A. fumigatus*, which was also confirmed by histological examination [36]. Compared to non-infected animals, no significant radioactive signal could be observed in the lung region for all antifungal siderophore conjugates.

## 4. Discussion

Antimicrobial siderophores have been attracting the interest of scientists for many decades. First discovered as natural products of bacteria, so called sideromycins, they were investigated as antibacterial compounds, e.g., salmycin and albomycin for growth inhibition of *Streptococcus pneumoniae* and *Staphylococcus aureus* [39,40]. This natural concept of utilizing the iron acquisition system of bacteria to channel antibacterial molecules inspired the development of new synthetic compounds. For example, pyoverdine (siderophore) was conjugated with ampicillin (beta-lactam antibiotic) against *Pseudomonas aeruginosa* [41]. The big advantage of the synthetic strategy is the high variability of conjugation partners. It is possible to use these siderophores as a “Trojan horse” to smuggle all kinds of molecules into bacteria [42]. The recently approved drug Cefiderocol (Fetroja^®^) by the FDA (November 2019) is the first artificial sideromycin on the market consisting of a catechol-chelating unit and a cephalosporin antibiotic (beta-lactam) against Gram-negative bacteria [43].

This Trojan horse concept can be adopted for fungal species as well, using fungal-specific siderophores to deliver antifungal molecules. Previous research published by our group showed that fluorescence dyes could be attached to [Fe]DAFC and a specific fluorescence signal could be detected in *A. fumigatus* hyphae that was dependent on active uptake via MirB [18]. Furthermore, “large” antifungal peptides were attached to [Fe]DAFC to inhibit fungal growth, but unfortunately, no antifungal effect could be observed [34]. In this study, we attempted to modify TAFC with small organic molecules that have known antifungal properties described in the literature and to label them with gallium-68 to perform PET/CT images of *A. fumigatus* lung infections in a rat model.

Synthesis of the antifungal siderophore conjugates was straightforward, with high purity of the resulting compounds. Modifications had to be made for eflornithine to provide better synthetic yields with a lower rate of side products. Fludioxonil had to be chemically modified by adding a linker function to its molecular structure for coupling to DAFC. The new compounds were labeled with gallium-68 to perform in vitro and in vivo tests. Labeling resulted in a high radiochemical purity under mild conditions (10 min, room temperature, pH = 4.5).

Antifungal siderophore conjugates used within this research were produced in two different modifications: iron-containing ([Fe]DAFC) and gallium-containing ([Ga]DAFC) conjugates. The rationale behind using gallium instead of iron was to enhance targeting efficiency because the iron transported by siderophores leads in *A. fumigatus* after uptake to downregulation of siderophore transporters such as MirB [44]. This would lead to a reduction or even cessation of siderophore uptake into the hyphae, and in the case of antifungal siderophore conjugates, result in a better survival of the fungus. Additionally, iron as an essential nutrient has a growth-enhancing effect on fungi. Gallium nitrate is a FDA-approved drug, used in cancer-related hypercalcemia (Ganite^®^), and showed a very good tolerance in patients [45]. It should be considered that gallium itself has an antifungal effect against microorganisms [46]. Bastos et al. have shown that Ga(NO_3_)_3_ has an antifungal effect on *A. fumigatus* cultures in different media [38], and Ga(NO_3_)_3_ is also currently investigated against *Pseudomonas aeruginosa* lung infection of adults with cystic fibrosis in a clinical trial [47]. So far, it is not completely clear how gallium influences the antifungal activity of the described siderophore conjugates, but MIC tests with [Ga]TAFC showed an inhibition of growth at 8 µg/mL and 24 h, but after 48 h, no inhibition was observed anymore. Fungistatic behavior of Ga(NO_3_)_3_ has also been described by Bastos et al. in AMM [38]. As seen in Figure 4, an overall better fungal growth inhibition was observed for gallium-complexed conjugates in comparison to iron-containing counterparts. In the development of antimicrobial siderophore conjugates as the “Trojan horse”, Miller, Nolan, and others, who generated fundamental knowledge in antibiotic conjugates, always used iron-chelated compounds [48,49,50,51]. One of the first reports on enhancing antibacterial potency by complexing gallium was published by Pandey et al. with Ferrichrome-Ciprofloxacin against *Escherichia coli*. They observed a 2–4-fold enhancement of activity compared to the iron complex [52].

Antifungal susceptibility assays revealed the potential of fungal growth inhibition by the produced antifungal siderophore conjugates. To test their MirB transporter dependency, two different media were used: iron-depleted AMM Fe(-) (upregulation of MirB transporter,) and iron-sufficient media AMM Fe(+) (control, no active transport). Except for [Ga]DAFC-eflornithine and –thiomersal, all gallium-chelated compounds showed a MirB-dependent antifungal effect. Thiomersal conjugates revealed an overall low MIC value independent on the media and containing gallium or iron, which is probably due to cleavage outside the hyphae resulting in ethylmercury (active metabolite) causing growth inhibition. Higher instability in human serum and in vivo fortifies these assumption but this limits the applicability, because adverse health effects of ethylmercury are still controversially discussed [31]. [Ga]DAFC-fludioxonil (16 µg/mL), -FOA (16 µg/mL), and -Cy5 (16 µg/mL) showed a growth reduction of 3 to 4 dilution steps in iron-depleted media compared to iron-sufficient media after 24 h, giving a proof of principle for a MirB-dependent antifungal effect. Eflornithine revealed no antifungal effect independent of the siderophore conjugation.

Competition assay with iron-containing antifungal siderophore conjugates blocking the uptake of [^68^Ga]Ga-TAFC showed a specific interaction of all conjugates with the MirB transporter, encouraging the hypothesis of a transporter-dependent effect. Uptake assays of [^68^Ga]Ga-DAFC-fludioxonil, -thiomersal, -FOA, and -Cy5 showed a high accumulation in *A. fumigatus* hyphae; however, the uptake of Cy5 could not be blocked by [Fe]TAFC or iron-sufficient media. This unspecific binding was already observed in earlier studies and origins probably due to interaction with the outer cell compartment [18,19]. Uptake of [^68^Ga]Ga-DAFC-eflornithine was notably low, which could be one reason why no antifungal effect was observed when coupled to [Ga]DAFC. Growth assays confirmed utilization of DAFC-eflornithine, -fludioxonil, and -FOA, whereas [Fe]DAFC-Cy5 and -thiomersal did not promote growth, providing limited additional information towards the theranostic properties of these compounds.

Beside growth-inhibiting properties, pharmacokinetics of these novel antifungal siderophore conjugates should be especially considered, which could predict diagnostic and therapeutic performance [53]. PET/CT images of non-infected rats show the distribution of a radioactive-labeled compound and the main excretion routes supported by the biodistribution results. [^68^Ga]Ga-DAFC-eflornithine and -FOA were mainly rapidly excreted through the kidney and bladder of the experimental animals. These findings are in line with a low protein-binding capacity and a hydrophilic character of the compounds. In a diagnostic setting, a rapid elimination from the body lowers radiation burden for the patient and allows for high image resolution. On the other hand, bioavailability for a therapeutic effect is reduced. Lipophilic compounds like [^68^Ga]Ga-DAFC-fludioxonil, -thiomersal, and -Cy5 were mainly excreted through liver and intestines and showed higher blood levels. This would decrease the diagnostic performance; however, a therapeutic effect could be enhanced due to longer circulation times, increasing the likelihood of interaction with MirB, and thereby uptake. Further important is stability, which, e.g., was reduced for [^68^Ga]Ga-DAFC-fludioxonil in vivo as compared to in vitro experiments with plasma, potentially leading to a lower concentration of active compound in circulation. The overall ideal pharmacokinetic properties of a compound are, however, also dependent on the mechanism of action of the antifungal conjugate, e.g., fungicidal compounds with higher toxicity may favor rapid pharmacokinetics, whereas a fungistatic mode of action rather would benefit from a slower elimination with the potential of prolonged action.

Diagnostic applicability of these compounds was tested in an *A. fumigatus* lung infection rat model. All antifungal siderophore conjugates revealed uptake into the infected area of the lung and a visualization in PET/CT. This specific accumulation holds promise for a therapeutic effect in vivo. Obtained imaging data were comparable with previous *A. fumigatus* infection images with [^68^Ga]Ga-TAFC by Petrik et al. and Skriba et al. [16,36,54] and modified [^68^Ga]Ga-TAFC by Kaeopookum et al. [17]. PET/MRI pictures by Henneberg et al. using the Aspergillus-specific antibody hJF5, labeled with copper-64 to visualize an *A. fumigatus* infection, also showed similar patterns in a mouse model [55].

Taken together, antifungal siderophore conjugates synthesized within this study are highly interesting model compounds for theranostic applications in fungal infections. The concept of using siderophore conjugates for theranostics, combining gallium-68 for PET with a therapeutic moiety, were first described by Ferreira et al. [56]. They coupled siderophore catecholate binding moieties to a DOTAM scaffold, and additionally ampicillin for antibacterial activity. The iron-containing compound did not reveal antibacterial activity in wild-type *E.coli*, but specific in vitro uptake could be proven. In vivo data of the therapeutic conjugate have not been provided. More recently, Pandey et al. [52] reported a siderophore ciprofloxacin conjugate based on ferrichrome. They showed a siderophore iron transport-dependent internalization of one compound and antibacterial activity against *Peudomonas aeruginosa*, *Staphylococcus aureus*, and *Klebsiella pneumoniae* of the Gallium (III) complex, whereas the Ferric complexes remained inactive, similar to our findings. They also reported biodistribution data in normal mice, but no data in animal infection models. Both studies also failed to provide proof of a therapeutic effect in respective infection models. Our study confirmed this principle using antifungal siderophores conjugates. We provided for the first time, specific in vivo targeting of infections with such “Trojan horse” conjugates by PET imaging and could show that the antifungal efficacy in most compounds was clearly related to the siderophore transport target MirB. However, in vivo treatment experiments have not been conducted yet and should be performed after improving the antifungal activity of this type of compound. This will allow us to fully exploit the possibility of antifungal siderophore conjugates as theranostic compounds.

## 5. Conclusions

In this study, we successfully modified TAFC with a variety of antifungal molecules with divergent mechanism of action, unrelated to commonly used therapeutics. Targeting the iron acquisition system of *A. fumigatus* with these antifungal siderophore conjugates revealed a proof of principle to utilize MirB for delivering a variety of antifungals to inhibit fungal growth in antifungal susceptibility tests. By labeling the compounds with gallium-68, a lung infection of *A. fumigatus* in a rat model was confirmed, also revealing insights about the influence of the conjugation partners on pharmacokinetic properties. Keeping in mind that antifungal resistance is an uprising problem, the MirB transporter as a target, which is essential for virulence of *A. fumigatus*, shows high potential for future applications. Knowledge gained within this research could lead to sophisticated strategies in the field of IPA infections, combining imaging and therapy in a theranostic approach.

## Figures and Tables

**Table 1:** Distribution coefficient, protein binding, and serum stability of siderophore compounds radiolabeled with gallium-68. Values of serum stability reflect percentage of intact radiolabeled conjugate. Results reflect three individual experiments; presented as mean ± SD. (*) Data from [17]; (**) Data from [19].

		[^68^Ga]Ga-TAFC *	[^68^Ga]Ga-DAFC-Eflornithine	[^68^Ga]Ga-DAFC-Fludioxonil	[^68^Ga]Ga-DAFC-Thiomersal	[^68^Ga]Ga-DAFC-FOA	[^68^Ga]Ga-DAFC-Cy5 **
Distributioncoefficient	LogD (pH 7.4)	−2.08 ± 0.02	−3.45 ± 0.04	1.30 ± 0.02	0.25 ± 0.06	−2.66 ± 0.01	1.03 ± 0.10
Protein binding [%]	30 min	2.54 ± 1.01	11.4 ± 4.8	14.6 ± 4.3	67.4 ± 2.7	2.4 ± 0.4	13.7 ± 2.9
	60 min	3.55 ± 0.68	8.8 ± 0.7	16.8 ± 5.1	71.1 ± 0.8	2.6 ± 0.7	13.1 ± 2.3
	120 min	2.96 ± 0.33	10.1 ± 1.9	15.0 ± 2.6	68.2 ± 1.2	3.3 ± 1.1	13.7 ± 1.8
Serum stability	60 min	99%	98%	96%	72%	98%	99%
	120 min	99%	99%	95%	87%	98%	97%
	240 min	99%	99%	95%	80%	99%	97%

**Table 2:** In vivo stability of different antifungal siderophore conjugates radiolabeled with gallium-68. Samples form BALB/c mice 10 min after injection into the tail vain. Values reflect intact conjugates measured by radio-HPLC analysis. (Each value represents two biological replicates).

	[^68^Ga]Ga-DAFC-Eflornithine	[^68^Ga]Ga-DAFC-Fludioxonil	[^68^Ga]Ga-DAFC-Thiomersal	[^68^Ga]Ga-DAFC-FOA	[^68^Ga]Ga-DAFC-Cy5
Blood	98.0%	40.6%	26.7%	>99.0%	>99.0%
Urine	99.0%	9.8%	30.5%	>99.0%	5.2%

**Table 3:** Biodistribution of gallium-68-labeled antifungal compounds in standard BALB/c mice. Injected into the tail vein and measured after 45 and 90 min, shown as injected dose per gram tissue (%ID/g). Important values are highlighted in bold. (Each value represents three biological replicates and presented as mean ± SD).

Organ	[^68^Ga]Ga-DAFC-Eflornithine		[^68^Ga]Ga-DAFC-Fludioxonil		[^68^Ga]Ga-DAFC-Thiomersal		[^68^Ga]Ga-DAFC-FOA		[^68^Ga]Ga-DAFC-Cy5	
	45 min	90 min	45 min	90 min	45 min	90 min	45 min	90 min	45 min	90 min
Blood	0.93 ± 0.63	0.13 ± 0.03	0.17 ± 0.06	0.14 ± 0.02	**10.35 ± 3.35**	**3.21 ± 0.94**	1.30 ± 0.62	0.13 ± 0.04	**4.38 ± 1.02**	**3.37 ± 0.65**
Spleen	0.38 ± 0.05	0.22 ± 0.01	0.32 ± 0.06	0.30 ± 0.05	1.82 ± 0.64	0.61 ± 0.12	0.33 ± 0.08	0.12 ± 0.04	1.56 ± 0.15	5.58 ± 0.94
Pancreas	0.31 ± 0.07	0.06 ± 0.01	0.05 ± 0.01	0.04 ± 0.00	1.59 ± 0.61	0.45 ± 0.09	0.28 ± 0.04	0.06 ± 0.02	0.56 ± 0.07	0.46 ± 0.12
Stomach	0.28 ± 0.06	0.09 ± 0.03	0.94 ± 1.12	0.25 ± 0.22	1.77 ± 0.50	0.72 ± 0.24	0.40 ± 0.07	0.13 ± 0.07	3.95 ± 3.13	1.77 ± 1.61
Intestine	0.68 ± 0.08	0.51 ± 0.05	**23.08 ± 2.43**	**25.19 ± 1.18**	**19.41 ± 7.26**	**13.63 ± 1.37**	2.68 ± 0.54	2.52 ± 0.40	**15.82 ± 0.31**	**24.73 ± 0.34**
Kidneys	**23.01 ± 2.23**	**19.35 ± 4.55**	0.22 ± 0.03	0.20 ± 0.03	**7.43 ± 2.24**	**3.67 ± 0.98**	2.72 ± 0.21	1.75 ± 0.21	**4.60 ± 0.52**	**3.37 ± 0.68**
Liver	0.55 ± 0.03	0.38 ± 0.05	2.20 ± 0.49	0.83 ± 0.23	3.67 ± 0.38	2.37 ± 0.83	0.70 ± 0.34	0.60 ± 0.38	**19.73 ± 2.25**	**22.95 ± 1.57**
Heart	0.28 ± 0.07	0.07 ± 0.01	0.07 ± 0.02	0.06 ± 0.02	3.03 ± 0.91	0.97 ± 0.14	0.33 ± 0.09	0.07 ± 0.00	1.39 ± 0.11	1.25 ± 0.25
Lung	0.89 ± 0.16	0.24 ± 0.04	0.32 ± 0.26	0.18 ± 0.09	7.04 ± 2.67	2.96 ± 1.75	0.66 ± 0.02	0.21 ± 0.06	2.95 ± 0.20	1.95 ± 0.25
Muscle	0.33 ± 0.23	0.04 ± 0.01	0.04 ± 0.01	0.03 ± 0.01	1.25 ± 0.49	0.36 ± 0.10	0.20 ± 0.02	0.05 ± 0.02	0.50 ± 0.12	0.45 ± 0.11
Femur	0.21 ± 0.09	0.11 ± 0.04	0.07 ± 0.02	0.09 ± 0.06	1.50 ± 0.72	0.56 ± 0.22	0.45 ± 0.17	0.14 ± 0.03	0.80 ± 0.43	0.85 ± 0.13

## Data Availability

Not applicable.

## Supplementary Materials

The following are available online at https://www.mdpi.com/article/10.3390/jof7070558/s1, Figure S1: 1H-NMR spectrum of ethyl fludioxonil-butyrate, Figure S2: 1H-NMR spectrum of fludioxonil-butyric acid, Figure S3: Microscopic images of A. fumigatus MIC assays with antifungal siderophore conjugates in iron depledet AMM, Figure S4: Microscopic images of A. fumigatus MIC assays with antifungal siderophore conjugates in iron sufficient AMM, Table S1: Overview of data included in this study.
